# Online questionnaire development: Using film to engage participants and then gather attitudes towards the sharing of genomic data^☆^

**DOI:** 10.1016/j.ssresearch.2013.12.004

**Published:** 2014-03

**Authors:** A. Middleton, E. Bragin, K.I. Morley, M Parker, on behalf of the DDD Study

**Affiliations:** a Wellcome Trust Sanger Institute, Cambridge, UK; b Addictions Department, Institute of Psychiatry, King’s College London, UK; c Centre for Molecular, Environmental, Genetic and Analytic Epidemiology, Melbourne School of Population and Global Health, The University of Melbourne, Melbourne, Australia; d The Ethox Centre, Department of Public Health, University of Oxford, UK

**Keywords:** Genome, Ethics, Survey, Incidental finding, Film, Opportunistic

## Abstract

How can a researcher engage a participant in a survey, when the subject matter may be perceived as ‘challenging’ or even be totally unfamiliar to the participant? The Genomethics study addressed this via the creation and delivery of a novel online questionnaire containing 10 integrated films. The films documented various ethical dilemmas raised by genomic technologies and the survey ascertained attitudes towards these. Participants were recruited into the research using social media, traditional media and email invitation. The film-survey strategy was successful: 11,336 initial hits on the survey website led to 6944 completed surveys. Participants included from those who knew nothing of the subject matter through to experts in the field of genomics (61% compliance rate), 72% of participants answered every single question. This paper summarises the survey design process and validation methods applied. The recruitment strategy and results from the survey are presented elsewhere.

## Highlights

• We created a novel, online survey including 10 short films.
• The extensive survey validation process involved 19 iterations before the final survey was ready.
• Focussing on the survey design paid dividends in high response rate and low drop out rate.
• Complex subject matter was no barrier to participant involvement.
• Using a film-survey combination was a successful strategy in terms of recruitment.

## 1 Introduction

The aim of the Deciphering Developmental Disorders (DDD) research project is to identify new genetic causes for developmental disorders in children (Firth et al., 2011). This involves testing children and their parents, using ‘exome sequencing’,^1^ a technique in which each of the individual’s 20,000 genes are explored – in this case, to uncover an explanation for the child’s disability.

The process of examining every gene offered by exome sequencing, also affords the opportunity to look at genes that are not known to be related to the child’s disability. The ethical dilemma here is whether to take this opportunity or not and what to do with any findings; such a dilemma can apply both to clinical sequencing in a health service and also sequencing in a research setting. The advantage of taking the opportunity to screen the exome or genome, as a whole, is that findings unrelated to the original investigation (in the case of the DDD, this was concerned with developmental disorders) could still be very relevant to the child in later life as well as to other family members. Such, ‘incidental findings’ could, for example, offer information about life-threatening and serious conditions. In the DDD research project, the decision was made not to seek findings other than those relating to the developmental disorder under investigation. However, we were interested in carrying out some complementary social scientific research – the Genomethics Study (Middleton et al., 2013) - to get a better understanding of stakeholder views on this important topic and uncover attitudes towards sharing of data from research sequencing projects.

Pressure is mounting from policy makers and ethicists for those who use whole genome approaches to share clinically significant incidental findings, the thinking being that it is unethical to withhold genetic information that could enable the research participant to take preventative or therapeutic action to protect their health (Affleck, 2009; Evans and Rothschild, 2012; Wolf, 2012). However, some genomic researchers are concerned that the time spent searching for, interpreting and reporting incidental findings unrelated to the research aims might jeopardise attaining those aims. Many health professionals are concerned about how such data would be managed and used in clinical practice and the potential difficulties this might generate. Empirical data is required on the attitudes of multiple stakeholders affected by and working with incidental findings and policy is urgently needed to guide researchers on what to do in terms of incidental genomic findings (Wright et al., 2011). Our study aims to gather empirical data and inform such policy.

The objectives of this research are to ascertain the attitudes of multiple stakeholders, i.e. members of the public (who could be recipients of genomic data), genetic health professionals (who would deliver the genomic data), genomic researchers (who would create the genomic data) and other health professionals (who would also interact with members of the public who have received genomic data). We aimed to investigate the following:

(1) Attitudes towards sharing of ‘pertinent findings’ from whole genome studies.
(2) Attitudes towards sharing of ‘incidental findings’ from whole genome studies.
(3) Attitudes towards receiving genetic information in different categories.
(4) Attitudes towards risk perception.
(5) Attitudes towards the sharing of raw genomic data.
(6) Attitudes towards genomic researchers having a duty to search for incidental findings.
(7) Attitudes towards who might filter genomic data.
(8) Attitudes towards possible consenting procedures for genomic studies.
(9) Socio-demographic information and how this might affect the above variables.

Each of the above themes considered complex ideas about what could be learnt from a genome or exome sequence and required a superficial level of understanding about genetics. Thus, particularly for the lay members of the public, it was important to ensure these were carefully explained in lay language. Film was used as the medium for this.

This methods paper summarises the questionnaire design process that was undertaken to develop and implement an online survey, which aimed to introduce, in an unbiased way, the ethical dilemmas raised by genomic technologies and ascertain attitudes towards these. The challenge in this was to create a questionnaire that was suitable for completion by a wide range of people. Thus, it needed to be engaging and interesting enough for participants who knew nothing of the subject matter as well as those who were experts in the field. This paper considers the extensive design process that was completed in order to meet this remit.

### 1.1 Overview of the genomethics study

The study adopted a mixed methods approach, utilising both quantitative and qualitative techniques. Non-parametric data was collected through 32 closed questions and analysed using descriptive statistics.

Throughout 2011–2013 a link to the online questionnaire was made available; the Wellcome Trust Sanger Institute in Cambridge, UK hosted the survey and associated website. Numerous recruitment methods were used to invite survey completion, including national media reports (e.g. coverage on the UK Channel 4 and BBC news), direct invitation from health professionals participating in the molecular DDD study through to advertisement of the study via Facebook, Twitter, a Genomethics blog and through Google ads. As the survey was online it had the capacity to be retrieved by anyone who had access to the Internet and thus also by people who had not received a direct invitation from the researchers. The complete recruitment strategy is discussed in more detail elsewhere (Middleton et al., 2014). The aim was to specifically recruit research participants who were genomic researchers, genetic health professionals, other health professionals and members of the public. Research Ethics Committee approval was granted for the study by the National Research Ethics Service for the UK (REC reference: 11/EE/0313).

The online questionnaire can be accessed at www.genomethics.org; although active online recruitment ceased on 16th July 2013 the survey can still be viewed on the website. This paper summarises the questionnaire design process that was completed to create a bespoke questionnaire. The survey used 10 films that described some of the ethical dilemmas raised by genomic technology; film was specifically chosen as a medium that could be easily incorporated in an online setting and also a tool capable of inspiring participants to engage with the study and to give them the introductory information they needed in order to be able to answer the questions.

No results from the survey itself are presented here, only the results pertaining to how the questionnaire was designed and validated. Information about the qualitative arm of the study, the social media recruitment strategy as well as the survey results will be published separately.

## 2 Material and methods

### 2.1 Stakeholders in the research

**Fig. 1:**
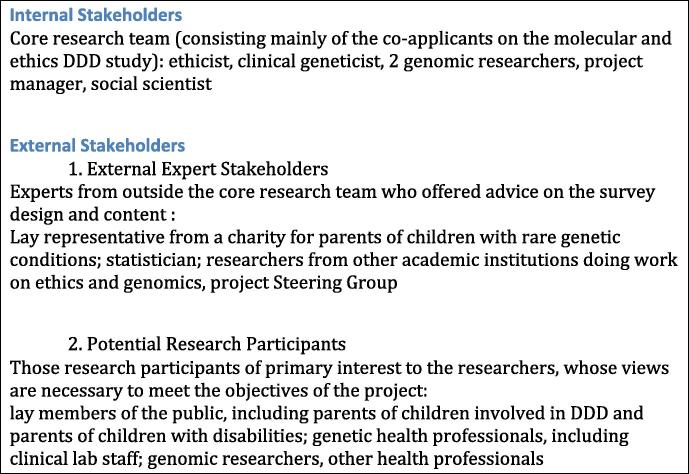
Stakeholder groups.

There are multiple stakeholders who were involved in the design of the research, including those who are part of the core molecular and ethics research team (‘Internal Stakeholders’); those who are connected to the research through the Steering Group or through academic interest (‘External Expert Stakeholders’) as well as potential research participants actively being recruited into the study (‘Potential Research Participants’).^2^ Input was sought in the questionnaire design process from all of the stakeholder groups, see Fig. 1 for details.

The final study sample, ascertained via the survey, needed to consist of four different groups of participants: members of the public, genetic health professionals, other health professionals and genomic researchers.

#### 2.1.1 Creation of the questionnaire

**Fig. 2:**
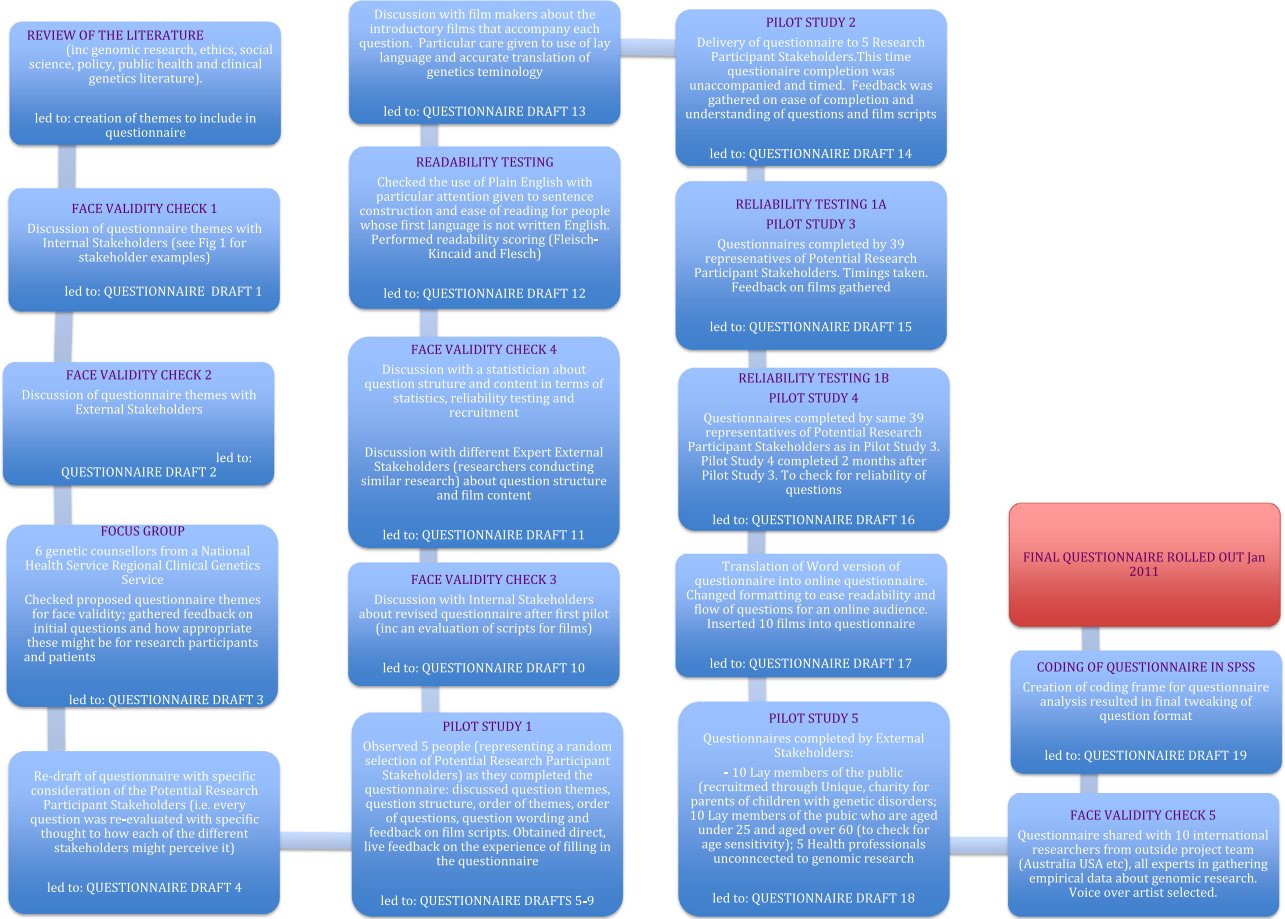
Validation process.

AM (Anna Middleton, lead author) led on the questionnaire design, conducted the Focus Group, pilot studies and face validity testing. She iteratively moulded, tested and retested the survey in order to create the survey and film scripts (see Fig. 2). EB (Eugene Bragin, second author) did the web based coding to turn the Word version of the questionnaire into a bespoke online version. An external film production company (www.neonotter.com) were contracted to work with AM; together they turned the validated scripts into film. The survey underwent 19 major iterations before it was considered ready for use.

Fig. 2 summarises, in a flow diagram, how the final questionnaire was created, detailing the validation process.

#### 2.1.2 Literature review

The questionnaire creation began with a thorough review of the literature, conducted between 2010 and 2011. The databases Pubmed and Scopus were used, with a combination of the search terms ‘genomic, incidental finding, research study, whole genome study, GWAS, ethics, results sharing, data sharing’. The literature review process formed part of the content validity of the survey.

#### 2.1.3 Face validity testing

The questionnaire was created using a systematic approach that adhered to robust principles of questionnaire design (Dillman, 2000; Denscombe, 2005; Aday, 2006; Lietz, 2010; Vicente and Reis, 2010). Each question was meticulously analysed, for content and understanding with the following issue at the front of the researchers mind: ‘will this question adequately assess what we want it to?’ The questionnaire was subjected to five formalised face validity checks, with all the Stakeholder groups as shown in Fig. 1. After each face validity check, feedback was gathered qualitatively (not measured quantitatively) and the survey was refined in response to stakeholder feedback. The face validity testing was interspersed between pilot studies; we deliberately structured it in this way so that any changes made to the survey items in response to the pilot work could be checked as appropriate with the stakeholder groups. Feedback from the pilot studies enabled the questions that started off open-ended to become refined into closed containing a short list of possible responses that participants could choose. The closed selection of answers could then be checked as reasonable within the face validity testing.

#### 2.1.4 Focus group

A focus group was conducted with informed external stakeholders (a group of six practicing genetic counsellors from a National Health Service Regional Clinical Genetics Service). Genetic counsellors were chosen because they are health professionals directly involved in recruitment into genetic and genomic research studies but also they work directly with members of the public. They have a wealth of experience in translating genetic language into lay language. AM took notes, which drew out themes representative of the discussions, these formed the basis of the outcomes.

#### 2.1.5 Pilot studies

A total of five pilot studies were completed; after each pilot the survey was refined in response to the findings. As the survey evolved it was tested and re-tested in the subsequent pilot studies.

**Pilot study 1** involved an observation of 5 potential research participants (1 internal stakeholder and 4 external stakeholders: 1 member of the public and 3 genomic researchers) as they completed the survey; participants were asked to provide a running commentary on what they were thinking as they worked their way through the survey. AM prompted participants so that feedback could be gathered on the following: (a) question themes, (b) question structure (e.g. prefer to answer yes/no or tick boxes on a Likert scale) (c) order of themes (d) order of questions within themes (e) question wording (f) feedback on film scripts.

**Pilot study 2** involved the delivery of the survey to a different set of 5 potential research participants (2 genomic researchers and 3 members of the public). This time the survey was not observed, but was timed. After completion, AM gathered feedback on the ease of completion of the survey and understanding of the questions and film scripts.

**Pilot study 3** involved the delivery of the survey to 39 potential research participants (all external stakeholders: 13 genomic researchers, 9 members of the public, 9 NHS clinical lab staff, 8 genetic health professionals) and then the delivery of the same survey to the same 39 potential research participants two months later (**Pilot study 4**). Participants were asked to offer feedback on the questions, ease of understanding and say how long the survey took. Responses from Pilot study 3 and 4 were compared via test–retest reliability testing.

**Pilot study 5** involved the delivery of the survey to 25 external stakeholders: 5 health professionals unconnected to genomic research and 20 lay members of the public recruited through the charity Unique (some of whom had previously had genetic testing and all of whom had a family history of a genetic condition), 10 of these participants were either aged under 25 or over 60 (to check that the questions could easily understood and completed by younger and older participants).

#### 2.1.6 Readability testing

Between pilot study 1 and 2 the survey was tested for readability using the opensource website, www.readability-score.com. This was done with a particular focus on the use of Plain English, i.e. the sentences needed to be shortened and edited to enhance clarity and understanding.

#### 2.1.7 Reliability testing

Test–retest reliability calculations were done between Pilot Studies 3 and 4, which were administered two months apart. As the responses to the questions are nominal the use of a kappa statistic, which compares the observed and expected amount of agreement for an item measured at two time points, was considered. However, kappa is dependent upon the proportion of the sample endorsing a particular response for an item; if the majority of individuals endorse the same response then the expected proportion of agreement will be large, and the estimate of kappa will be very low despite high observed agreement levels (Feinstein and Cicchetti, 1990; Brennan and Silman, 1992). As we expected a large proportion of the sample endorsed the same item for each question, we therefore only calculated the observed level of agreement per question. We also evaluated the stability of responses over time by asking participants to leave their email addresses at the end of the survey if they were amenable to being contacted and asked to repeat the survey again over a year later. However, as this wave of data collection was not designed to inform questionnaire development, we do not report the results here.

#### 2.1.8 Turning a word version of a survey into an online version

**Fig. 3:**
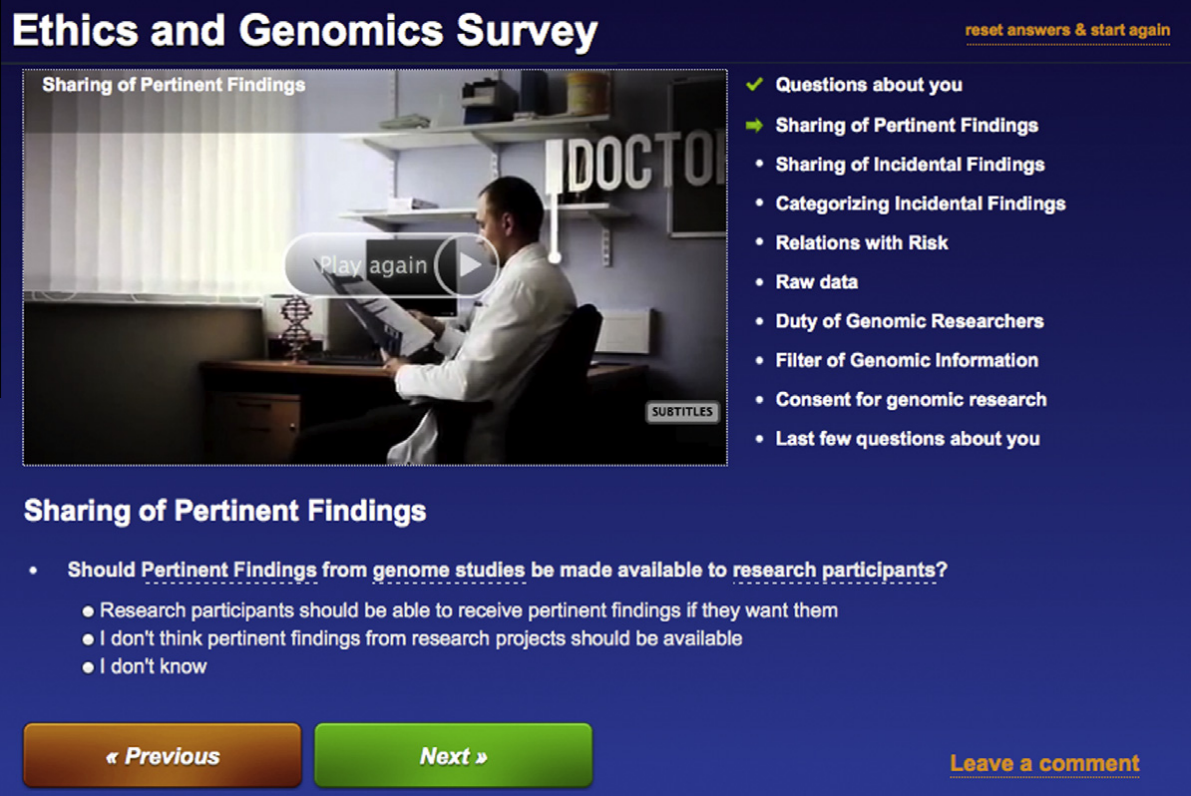
Screenshot showing the visual layout of the online survey.

The online questionnaire was bespoke and a web programmer created coding specifically to optimise the usability. In accordance with good practice for web-based questionnaire design, in-depth consideration was given to the online style of the questionnaire (Vicente and Reis, 2010). For example, there is evidence to suggest that participants in an online questionnaire make an initial assessment of how many questions there are and how long they perceive it will take them to complete, if they cannot immediately make this assessment then they are less motivated to continue and may decline participation (Ganassali, 2008). Thus the online questionnaire was deliberately formatted so that it was easy to see how many sections there were, and particular attention paid to making the navigation experience easy so that participants could see how much progress had been made as they were working through the questions (via a permanent menu on the right hand side that lists all the themes in the questionnaire, a tick appears as each section of questionnaire is completed) (Vicente and Reis, 2010), see Fig. 3.

In addition to this there is evidence to suggest that there is less of a dropout rate and question omission rate if the questions are presented individually and the research participant has to actively click a button to reveal the next question as opposed to needing to scroll down the screen (Lozar Manfreda et al., 2002). Therefore, the questions were formatted using multiple pages, so that participants did not have to scroll down layers of text. The longer the online questionnaire, the higher the dropout rate (Ganassali, 2008), thus the questionnaire was designed to only take approximately 20 min to complete (considered ‘short’ in terms of online questionnaires).

To improve the user experience we added a glossary of terms so that if participants hovered their mouse over an underlined word a short description of the meaning of the word appeared in a box. We also made it possible to easily move forward and backward in the questionnaire so that participants could check previous results (we retained a footprint of when answers were changed so we could keep a record of this). As every result was stored, participants had the option of leaving the questionnaire at any time and coming back to it without having to start again from the beginning. This gave the optimum flexibility so that the questionnaire could be completed in multiple sittings if necessary. There was also a ‘reset and start again’ button so that a new user could complete the questionnaire from the same machine. This was particularly helpful for families or couples who might use one machine.

Online questionnaires that have a plain background have a lower dropout rate and higher completion rate than those with bright colours (e.g. purple and pink) (Dillman et al., 1998), therefore we chose a plain dark background with light text – this configuration is thought to support best practice formatting for visually impaired research participants. The questionnaire had also been checked to ensure that it was appropriate for an International audience – UK-centric words such as ‘GP’ were explained via a definition in the glossary and the socio-demographic data contained variables that were applicable to any participant, irrespective of geography. Consultation as to the worldwide accessibility of the language was sought by making comparisons to validated questionnaires used by the World Health Organization in 100 + different countries (e.g. http://www.worldvaluessurvey.org/index_surveys).

#### 2.1.9 Use of film

An independent film-maker was consulted from a London-based production company (www.neonotther.com). He met with AM on five separate occasions prior to filming to learn about the subject matter and discuss how best to translate this into film. Together, 10 scripts were developed, each of these subjected to evaluation via the validation process as detailed in Fig. 2. Visual metaphors were used to represent various themes, for example, the film exploring genomic incidental findings features two ‘doctors’ (white coats, looking at an X-ray) who are discussing what they have seen on the X-ray – this discussion simulates how a discussion might happen when genomic incidental findings are uncovered. Doctors and X-ray pictures are used here as this may be more of a familiar scene to lay members of the public than genomics (view supplementary figure).

The film scripts were subjected to two sets of independent face validity checks with both internal as well as external stakeholders as well as two pilot studies involving primary and secondary stakeholders (see Fig. 2 for details). Reviewers included a mixture of language users, some whose first language was British-, American- and Australian-English and others who used English as a second language. Each reviewer was asked to assess for understanding, readability and to anecdotally offer an opinion on whether the information being delivered was neutral and unbiased. The wording in the survey was adapted according to this feedback.

The films were overlaid with the following:

1. Subtitles (to aid participants with hearing impairment, participants whose first language was not English and support those who respond better to visual messages).
2. Voice over (to aid participants with visual impairment as well as those who respond better to auditory messages).
3. Animation (to help spell out particular items that could not be represented easily in film, e.g. showing a DNA double helix).

The voice over artist in the films was chosen for his ‘reassuring but authoritative’ manner (those who sounded high pitched or childlike were discounted); over 50 different voice over artists were considered; the film maker and first author narrowed down the choice to five artists and these were reviewed by an independent group of 10 researchers (Face Validity check 5, see Fig. 2) covering various nationalities: British, American, Canadian, Australian, Chinese, French, Swiss, Iranian. The independent group had the final decision on which voice-over artist they felt offered the most ‘reassuring but authoritative’ ambiance and this artist was chosen.

The background music on the film was carefully selected to be unobtrusive and ‘neutral’ in tone (not too ‘serious’ and possibly alarmist and not too light hearted and potentially frivolous). The music was changed several times after review with external stakeholders to make sure the ambiance was appropriate. The films were hosted by the Wellcome Trust Sanger Institute server and the finished questionnaire and integrated films can be found at www.genomethics.org.

## 3 Results

### 3.1 Response to the survey design process

#### 3.1.1 Literature review

From the literature review, broad themes were drawn that formed the basis of the questionnaire (Human Genetics Commission, 2000; Knoppers et al., 2006; Cho, 2008; Wolf et al., 2008; Beskow and Burke, 2010). These themes addressed two needs: (1) they covered issues that other researchers had identified as important for further study (2) they covered issues that policy makers had anecdotally identified as important for practice, but for which there was limited empirical data to support them. The themes were:

(a) The perceived duty of care of genomic researchers to research participants.
(b) What to do with incidental findings.
(c) Consent surrounding genomic information.
(d) Ownership of DNA and the information in it.
(e) How genomic data should be delivered.

#### 3.1.2 Face validity testing

The 5 themes (a–e above) established from the literature review were discussed with Internal Stakeholders (see Fig. 1) as part of the first face validity check, this resulted in the creation of a list of questions that could be attached to each theme. The list of themes and associated questions were then discussed with External Stakeholders (see Fig. 1) as part of the second face validity check. Face validity tests 3 and 4 were done after the first pilot study, the purpose of these was to touch base again with the internal stakeholders and also a statistician to double check that the direction the survey was taking was still in line with what they felt was appropriate. We also created a coding frame in SPSS (Software Package for the Social Sciences, IBM) and this resulted in the item format being updated slightly to make it easier to code. The face validity process helped to mould the themes from those presented above into the following ones here:

(a) Knowledge about genetics.
(b) Sharing of findings – pertinent and incidental.
(c) How to categorise incidental findings.
(d) Interest in sharing data with different risk levels.
(e) Attitudes towards sharing raw data.
(f) Duties of genomic researchers.
(g) How should genomic data be filtered and also delivered.
(h) Consent procedures.
(i) Socio-demographics.

The face validity testing that we undertook could also be considered as Content Validity as we took a subjective overview from experts in the field as to whether the items were appropriate or not in terms of their content.

#### 3.1.3 Focus group

The Focus Group discussion was initiated by AM and centred on a debate about the existing questionnaire themes and initial questions as defined at the beginning of the design process. The genetic counsellors were asked to explore whether, in their opinion, the themes and questions could be useful tools to gather data to address the study aims. They were also asked for their own opinions on the feedback of incidental findings. AM took notes that were later grouped into themes that were used to guide the next iteration of the survey questions. The following themes emerged:

• Film is an appropriate media for using in the survey.
  – The genetic counsellors agreed that it had the potential to appeal to a cross section of stakeholders, ranging from those who knew nothing about genomics through to those who had high knowledge levels.
• The themes and questions identified so far would address the study aims.
• Genetic health professionals should be gatekeepers of genetic information.
  – Not all genomic results should be fed back to research participants and genetic health professionals would be ideal professionals to make clinical decisions about what could be fed back and what could be withheld.
  – There is much pressure on genetic health professionals to manage the genomic findings from research and they resent this potential burden.
• There is a fear about how genetic health professionals will manage the outputs from a whole genome/exome sequence.
  – How should consent be constructed?
  – How should results be delivered (in clinic? Or via an indirect route such as through a website?)
• Incidental findings should categorised so as to manage the potential volume of data more easily.
  – Some categories of data should be made available (e.g. information relating to serious conditions that can be prevented) but not others (e.g. ancestry data).
  – The first iteration of the categorisation of IFs question was developed within the Focus Group. The categories that the genetic counsellors suggested included: conditions that are life-threatening or serious and cannot be prevented versus those that are life-threatening or serious and can be prevented; information relating to medication response; carrier status, information that is not immediately relevant but could be useful later in life; information that is uncertain and cannot be interpreted at the moment; information that is not of serious health importance; ancestry data.

#### 3.1.4 Pilot studies

The design process resulted in a quantitative questionnaire that consisted of 32 closed questions. The questions were short (Fink, 2003) and had fewer than 20 words per sentence (Oppenheim, 1992). The closed-end responses were listed vertically (Aday, 2006) and demographic questions positioned at the end (Lietz, 2010).

**Pilot study 1** The survey delivered in the first pilot study was paper-based and observed by AM as participants filled in each questions. Participants were asked to provide a running commentary on their experience of completing the questions. The following areas were discussed: (a) question themes – participants appeared to understand what the survey themes meant and also what was being asked of them in the questions; however, they did not immediately understand what was meant by the ‘perceived duty of care of genomic researchers’ theme. Members of the public in this pilot did not know that genomic researchers and health professionals may not have different duties of care. Thus questions under this theme were removed from the survey from this point on and this was checked as acceptable via the Face Validity check 3; (b) question structure – 4 out of 5 participants said they preferred to tick yes/no boxes rather than offer a rating on a scale, i.e. Likert scales were unpopular, thus from this point onwards they no longer appeared in the survey); (c) order of themes – participants preferred the socio demographic data at the end, but wanted to have some introductory questions at the beginning that asked who they were, therefore, the socio-demographics were split and labelled ‘questions about you’ so that the survey could be introduced with two easy questions that asked who participants were, then moving onto the body of the survey and ending with the remaining socio-demographics; participants said they wanted the two questions assessing knowledge of genetics to be delivered at the beginning of the survey as they found them a useful introduction to getting to think about what genetics was; (d) order of questions within themes – in the categorisation of incidental findings question, participants wanted to be asked the questions in a format that showed a perceived level of seriousness, i.e. information about life-threatening conditions that cannot be prevented should appear before information about ancestry; (e) question wording – 3 out of 5 participants felt that the questions were too long and cumbersome to read, feedback was offered on which questions needed to be shortened – in particular the ones on sharing pertinent and incidental findings and also the questions on the filter of data – we originally asked participants to state whether a *genetic counsellor* and *clinical geneticist* should be involved in the feedback of data, but 4 out of 5 participants said they were unsure if most people would know what these professions were, so we relabelled the groups as ‘health professional’ and ‘scientist’. We also initially designed the questions on raw data and filter of genomic data so that they were open, the responses given in Pilot study 1 to these open questions gave us a selection of plausible answers we could use in a closed set of responses in subsequent survey iterations; f) feedback on film scripts – all participants felt the information provided in the film scripts was pitched at too high a level and needed to be re-written more in lay language.

**Pilot study 2** The survey used in this pilot had been modified in response to the feedback from Pilot study 1 and was also paper-based. The second pilot involved timing how long it took 5 participants to complete the survey. Timings ranged from 30–60 min. The aim was for the survey to take 20 min or less and so the film scripts were shortened again, and several individual items were removed.

**Pilot study 3** and **Pilot study 4** The survey used in these pilot studies were paper-based. Feedback was taken on the shortened film scripts and understanding in lay language. Free text comments that were provided by participants indicated that the information provided in the film scripts were easily understood and helpful. Participants also reported that they felt the film scripts delivered information in an unbiased and neutral manner. The test–retest reliability results taken from these pilots studies are reported later.

**Pilot study 5** This was the first pilot study to use an online format. By this point the films had been created and so there was no possibility of changing the content within these; however we were able to alter the voiceover, music, subtitles and logistics with respect to how they were delivered. Participants were encouraged to offer their written feedback within the comments boxes on the experience of completing the survey online. Participants wanted the films not to start automatically when the survey opened, they wanted to be able to choose to click on the film when they were ready to view it. Participants said they really enjoyed the films: some comments – ‘I thought [the survey] was generally of a very high quality and the film clips were good’, ‘I really enjoyed this survey, it got me thinking, the films were brilliant’. We had used bullet symbols in the list of themes down the side of the survey, some participants thought they were radio buttons and tried to click on these buttons, so in response to this the bullets were changed to an arrow that turned into a tick on completion. The subtitles did not scale up when the screen was made larger and so we corrected this. Four participants reported that the background music was too loud in relation to the voiceover and so we rebalanced this so that the music was less intrusive. One participant also reported that the music on one of the films had a slightly ‘alarmist’ tone to it, so we changed the music on this one so that it was more similar to the others.

#### 3.1.5 Readability testing

The opensource website, www.readability-score.com, was used to assesses web-based text for readability scores. Our text was entered into the website and the calculations showed that there were, on average, 10.9 words per sentence and that The Fleisch-Kincaid Reading Ease score was 58.4 and Average Grade Level was 9.5, meaning that the text should be readable to a 15–16 year child. This was deemed by the internal stakeholders as an appropriate reading level for our survey.

#### 3.1.6 Reliability testing

For the 39 participants repeating the survey at one time point (Pilot Study 3) and then two months later (Pilot Study 4), the level of agreement between time points ranged from 0.68–0.95, with the majority of items scoring over 0.8. The question that scored 0.68 gathered attitudes towards the principle of sharing incidental findings in a research setting.

### 3.2 Response to the survey

For our survey we had 11,336 hits on the website. From these visitors, 4006 closed the survey without browsing further, i.e. 7330 continued to have a look at the site and begin answering the questions (indicating an initial 65% compliance rate). Three hundred and eighty-six records were removed as participants either dropped out after the third question or provided inconsistent answers. The first two questions asked if participants were health professionals or genomic researchers; the third question asked if the participant had ever been a research participant in a genetic research project; the fourth question asked participants to rate two statements about genetics as ‘true’ or ‘false’.

Of the remaining 6944 participants who answered more than the first three questions, >75% continued through the survey to the final ‘thank you’ message at the end and of these 4993 participants (72% of the final sample) answered every single question including the socio-demographic ones at the end. For each individual question more than 80% of participants provided an answer (i.e. there was, at most, missing values 20% of the time but for the majority of questions this was significantly less than 20%). Thus, the drop out rate (at any point in the survey) was very low.

From the 11,336 initial hits on the survey, 6944 participants were in the final sample, this gives an overall compliance rate to the survey of 61%.

From the final sample of 6944 participants each record was checked individually to ensure that the answers were consistent, we did this process by eye, going through each record at a time. The 386 records that were removed consisted of participants who answered the first two questions but then stopped at the third question. It also included participants who answered the first 3 questions but then missed out more than 75% of the remainder of the survey, with sporadic answered delivered and missing answers either side. For example in response to the questions in the section ‘Relations with Risk’ participants are asked to provide an answer to 4 different subsection. Participants were removed if they had seemingly given a sporadic answer, i.e. only answered one of the subsections, missed out the next 3 themes, then only answered a subsection of another theme. Also, amongst the 386 records that were removed were those where participants appeared to deliver a response set (Frankfort-Nachmias and Nachmias, 2000), i.e. where participants appeared to just provide one answer such as ‘yes’ or ‘don’t know’ to every single question.

Thus, once engaged in the study (i.e. passing the third question) subjects were motivated enough to participate in most of the survey. Whilst we did not specifically measure this, one could infer that participants understood what was being asked of them and the survey design enabled them to appropriately engage with the research.

Table 1 contains demographic data for the final sample. This shows that the four groups we set out to ascertain were successfully recruited (members of the public, genetic health professionals, other health professionals and genomic researchers). As a whole, the sample were most likely to be over the age of 31, female, highly educated, White and married. The significance of this in relation to attitudes towards the ethical issues surrounding genomics is discussed elsewhere. In addition to this an evaluation of the recruitment methods is also published separately.

## 4 Discussion

### 4.1 Paper or web?

Social sciences questionnaires have traditionally been paper based. It is only relatively recently that the questionnaire has taken an electronic form. Web based questionnaires have been evaluated in many studies and found to be acceptable to research participants (Kamo et al., 2011) and sometimes preferable to a paper version (Balter et al., 2005; Mathieu et al., 2012) not least to say, cost effective compared to printing and posting a paper questionnaire (Hunter et al., 2013). Interestingly, participants are more likely to complete an online questionnaire compared to a paper one (Balter et al., 2005; Kongsved et al., 2007) and whilst one might assume there could be a sampling bias between respondents to a postal questionnaire versus an equivalent online version, in one study that explored this issue, no statistical difference was found between these two methods and response rate and socio-demographic data provided (Fleming and Bowden, 2009). Other research has shown similar response rates between web-based and postal questionnaires (Truell et al., 2002; Ritter et al., 2004). We wanted to reach an International audience to ask views about a complex subject matter. On balance the easiest and most effective way to do this was via a multi-media approach on the Web.

### 4.2 Survey validation

As the study questionnaire was developed from scratch and – at least at the time of creation – no other work had been published of a similar nature there was no ‘gold standard’ against which to validate the individual items.

Socio-demographic questions were based on validated items used worldwide (World Value Surveys, 2009). The two questions that assessed knowledge about genetics were also previously validated items (Smerecnik et al., 2011). However as the rest of the survey gathered subjective attitude and opinion, cross-sectionally, it is very difficult to validate the attitude items using traditional methods. Thus, validation techniques that might be applied to, for example, a depression scale, do not apply to our questions. As research grows in the area of genomics and attitudes towards sharing incidental finding, and more researchers independently gather data on the same attitudes within and between different populations, it should be possible to compare our results (and thus questions) with other studies. This then enables the application of traditional validation techniques. As part of our own research we will be conducting interviews with all stakeholder groups. This will afford the opportunity to explore whether attitudes given in the survey match those given in the interviews. In addition to this, researchers from Denmark are also looking into translating the survey into Danish and ascertaining attitudes from a Danish population. This will again offer further opportunity to understand whether the questions adequately assess attitudes within a different population. Thus steps will be taken in the future to analyse whether the survey design was adequate. However, until this further work yields fruit, we had to create the survey as best we could, as happens within any exploratory and new area of research. Thus, the survey questions were created iteratively, after a 10-month consultation process (see Fig. 2) using input from all internal and external stakeholders (see Fig. 1 for details).

The reliability testing results are very encouraging. The test–retest calculations where a correlation over 0.8 is demonstrated, indicate that there is a strong level of agreement in responses to the same item at two different time points, the majority of our scores were over 0.8. One interpretation of this is that the items are a good measure of attitude. There was one correlation score that fell beneath the 0.8 cut off, this related to the generic question about sharing incidental findings, this appeared at the beginning of the survey and had a correlation of 0.68. In Pilot Study 3 participants were most likely to answer positively towards this, then in Pilot Study 4, 33% of those who had previously answered positively, changed their mind to ‘no’ or ‘don’t know’. This question appeared at the beginning of the survey and it is possible that as participants worked their way through the questions that they began to think more carefully about their attitudes, so that on the repeated survey they were more likely to answer cautiously.

### 4.3 Minimising bias

On first thought for many people (lay people as well as health professionals) genetics may appear to be a ‘difficult’ subject for which they feel ill equipped to understand (Farndon and Bennett, 2008). We know that many people have a media-influenced knowledge about genetics that is guided by what they see on the TV, Internet and in the newspapers (Bates, 2005; Condit, 2010). In addition to this, those who have a family history of a genetic condition can have their attitudes towards genetics influenced by factors explained by attribution theory (McAllister, 2010), family systems theory (Galvin and Young, 2010) and discourses aligned with society, expert and media views (Parrott et al., 2010). Knowledge exists within many forms in society and all forms are equally valid. However, when asked to give attitudes towards issues surrounding genetics there is no level playing field. Therefore, we wanted the films to deliver information simply – via different methods (words, imagery, animation, subtitles, voice-over, music etc.) – language that was not leading and raising ethical issues through questions rather than answers. The ultimate aim of this was to provide information in as many different formats as possible, without biasing the information in a particular direction. We wanted to know if participants felt biased or persuaded to answer in a particular way and asked this in the face validity testing. All answered not. Nothing more formalised was done in terms of measuring this.

The challenge of this project was to create a research tool that was engaging enough to capture the interest of potential participants, irrespective of their knowledge or understanding of genomics. We also required participants to deliver good quality data (no random button pressing) and to complete as many questions as possible. It is a common problem in any questionnaire-based research (web- and paper-based) for respondents to drop out part of the way through. The expression ‘mid-terminates’ refers to participants who reach a point of fatigue, boredom or do not perceive the survey of having personal value to them. If more than 75% of the participants stop the survey part way through and drop out (i.e. become mid-terminates) then it is important to take steps to improve the survey design (MacElroy, 2000). Compliance rates for individual surveys will be determined by the subject matter and the target population’s motivation to be involved.

### 4.4 The bespoke survey design process

We deliberately chose to apply the design process in the way we did because we wanted to create as many opportunities as possible to check that the survey was going to collect the data we needed to meet the study aims. Using a mixture of face validity testing and pilot studies offered multiple occasions to check, refine and re-check that the questions were appropriate for multiple stakeholders, one of the roles of the research is to ‘balance the demands of the different stakeholders’ (p8 Bruce, 2013). Thus on reflection about the applicability of the methods we used, we feel that they have been driven by the necessity to meet the demands of several very different stakeholder perspectives. If less stakeholders were involved it is highly likely that fewer pilot and face validity tests would have been necessary.

There are strengths and limitations to what we did in terms of survey design. The interplay of face validity testing with pilot testing on top of a focus group was incredibly helpful in enabling feedback from multiple stakeholders as to how the survey iterations were working. All of this work sat on the shoulders of the original literature review and was supported by the subsequent readability and reliability testing. However, what we were unable to do, due to time, size and resource constraints was additional statistical analysis of items at various stages in the design process. What would have been helpful is a preliminary analysis of associations between variables, for example we could have explored whether there was a bias from the order of the questions (Dillman, 2000). We could have also done much larger pilot projects, e.g. 100 participants or more and then had the ability to explore item nonresponse rate (Saris and Gallhofer, 2007) and whether there were specific biases shown in responses to particular variables.

## 5 Conclusion

A bespoke questionnaire that contained 10 integrated films was created to gather attitudes towards various ethical issues raised by genomic technology. Extensive validation work was conducted with multiple stakeholder groups, over a 10-month period, to iteratively develop the survey so that it would appropriately engage participants, from a whole range of backgrounds. The subject matter was totally unfamiliar to some participants (e.g. members of the public) and very familiar to others (e.g. genetic health professionals and genomic researchers). The film-survey approach was deemed successful in that we received 6944 surveys, 72% participants completed every question and more than 75% reached the final ‘thank you’ message at the end. Thus compliance with the survey was high. Survey results together with a more detailed description of the recruitment methods used are published elsewhere. We conclude that spending time on the design-phase of this study paid enormous dividends later in that a whole range of different participants appeared to be able to engage well with the study – enough to retain interest and complete most of the questionnaire. We were able to recruit large numbers of all the different participant groups we wanted and most importantly of all, we were able to gather useful and novel data from stakeholders that can be used to inform policy creation on the sharing of data from whole genome research studies.

## Figures and Tables

**Table 1:** Demographic data.

Variable	Subset of sample	Percentage of sample (*n* = 6944) (%)
Genetic Health Professionals	533	8
Other Health Professionals	843	12
Genomic Researchers	607	9
Members of the Public	4961	71
Total	6944	

*Age*		
Under 16	53	1
17–20	180	3
21–30	1174	22
31–40	1651	30
41–50	1115	20
51–60	685	13
61–70	474	9
71–80	111	2
81+	8	<1

*Gender*		
Male	1408	25
Female	4012	74
Prefer not to say	34	1

*Education*		
Completed primary school/preparatory school/elementary school	83	2
Currently studying at secondary school/high school	74	1
Completed secondary school/high school	719	13
Currently studying at university/college/other tertiary education institution	486	9
Completed degree(s) at university/college/other tertiary education institution	3798	70
Other	294	5

*Ethnicity*		
White	4974	92
Afro-European, African American, Black	52	1
Hispanic	60	1
South Asian Indian, Pakistani	112	2
East Asian Chinese, Japanese	60	1
Arabic, Central Asian	30	1
Other	163	3

*Marital status*		
Married/civil partnership/living together	3795	69
Divorced	271	5
Separated	85	2
Widowed	88	2
Single	1212	22

## Appendix A Supplementary material

Supplementary video 1 Video of one of the films from the survey.

## Footnotes

1 ‘Exome sequencing’ is the laboratory technique where the protein-coding regions of genes are examined. ‘Whole genome sequencing’ is the laboratory technique where the protein coding regions of genes, as well as the DNA between genes, are examined. Irrespective of the actual method used, the ethical dilemmas are the same with both techniques.

2 These stakeholders groups may be defined differently in the molecular DDD research; no attempt was made to link participants in the molecular study to those in the ethics study.
