# Supplementary figures and images for: Online questionnaire development: Using film to engage participants and then gather attitudes towards the sharing of genomic data
Source: Soc Sci Res. 2014 Mar;44(100):211–23. doi: 10.1016/j.ssresearch.2013.12.004 (PMC3969308; doi:10.1016/j.ssresearch.2013.12.004)

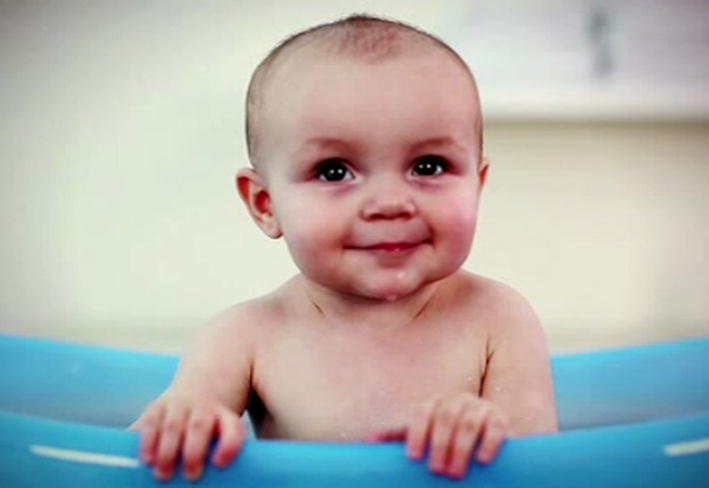

Supplement: Supplementary video 1 — Video of one of the films from the survey. [file mmc1.jpg]
